# Antimicrobial Activity of Bee Pollen: Influence of Botanical Origin and Processing

**DOI:** 10.17113/ftb.64.01.26.9421

**Published:** 2026-02-15

**Authors:** Tajda Lukman, Sonja Smole Možina

**Affiliations:** University of Ljubljana, Biotechnical Faculty, Department of Food Science and Technology, Jamnikarjeva 101, 1000 Ljubljana, Slovenia

**Keywords:** botanical origin, antimicrobial activity, exine, processing methods, bioavailability of active compounds, quality standardization of bee pollen

## Abstract

Bee pollen is a nutrient-rich bee product and natural food supplement that contains proteins, vitamins, minerals and bioactive compounds, offering antioxidant, anti-inflammatory, immunostimulatory and antimicrobial activity. Numerous studies have confirmed the *in vitro* antimicrobial activity of both polyfloral and monofloral bee pollen. Monofloral bee pollen had a more stable chemical composition and more consistent sensory and biochemical properties, making it more suitable for various applications. This has led to a growing number of studies investigating its antimicrobial potential. Antimicrobial activity of bee pollen is influenced by natural factors such as the botanical and geographical origin, seasonal variation and beekeeping practices. The outcomes of *in vitro* testing also depend on choices related to extract preparation, solvent type, microbial strains and the method employed to measure antimicrobial activity. Another challenge is the limited bioavailability of bioactive compounds, restricted by the degradation-resistant outer layer of bee pollen, named the exine. The wall can be partially disrupted through processing methods that break it and enhance its nutritional and functional properties. This review provides a comprehensive overview of published studies on the antimicrobial activity of monofloral bee pollen. It summarizes the most frequently investigated botanical species and bacterial strains, highlighting those with the most promising antimicrobial results. Additionally, it examines the processing methods of pollen, comparing their effectiveness and the changes in antimicrobial activity before and after processing. The review identifies the plant species, solvents and methods that yield strong antimicrobial activity, emphasizing their potential in the broader effort to standardize high quality parameters for bee pollen.

## INTRODUCTION

Consumer awareness of the impact of food on well-being is increasing, and the growing interest in natural products is driving this shift. Bee pollen was already recognized as a valuable nutritional source by the earliest civilizations, as evidenced by cave paintings in Spain. In antiquity, it was referred to as ‘the dust that gives life’ (*1*) and was attributed with therapeutic properties, playing an important role in religious rituals. However, its widespread use for human consumption began only after the Second World War (*2*).

Due to its rich nutritional composition – proteins, essential amino acids, carbohydrates, lipids, vitamins (primarily B group), carotenoids, minerals and polyphenols, bee pollen is a unique natural dietary supplement with high energy and biological value. It supports various physiological functions and strengthens the immune system through its bioactive properties, notably antioxidant, anti-inflammatory, immunostimulatory and antimicrobial activity (*3*, *4*). These effects originate from functional compounds such as phenolic acids, flavonoids and phenolamides (*5*-*7*). Today, bee pollen is also used in food as a natural preservative to prevent oxidation, enhance nutritional value, texture, taste and aroma, accelerate fermentation, and serve as a functional ingredient in meat products, dairy beverages, juices and bakery products (*8*).

The chemical composition of bee pollen is primarily determined by its botanical origin, *i.e.* the plant species from which the pollen is collected (*9*). The concentrations and diversity of phytochemical compounds vary considerably among species, and their specific chemical structures influence bioactivity, including antioxidant and antimicrobial effects (*5*). Additionally, bee pollen composition is affected by geographical location, season, weather conditions during collection, bee subspecies and beekeeping practices (*10*). Bees are highly selective when collecting pollen, usually foraging from one or just a few plant species at a time (*11*). However, environmental conditions often hinder the collection of monofloral bee pollen, so mostly polyfloral bee pollen is harvested. Polyfloral bee pollen varies significantly in plant-dependent chemical composition, nutritional value, and sensory, technological and functional properties (*12*, *13*). In contrast, monofloral bee pollen offers a more stable chemical composition, with consistent sensory and biochemical characteristics, making it more suitable for quality standardization and diverse applications (*14*).

Another challenge in the efficient use of bee pollen lies in the complex structure of the pollen wall, which significantly limits the release and bioavailability of its nutrients and bioactive compounds. This barrier reduces the absorption and utilization of beneficial substances, restricting the full nutritional and bioactive potential of bee pollen. The outer layer of the pollen wall, called the exine, is composed of sporopollenin, a highly resistant organic biopolymer. Together with the inner layer, known as the intine, and the membrane envelope, it protects the intracellular contents of the pollen grain from high temperatures, pressure, corrosion, wall degradation, and other environmental factors. A key focus in contemporary bee pollen research is the development of techniques to disrupt the pollen wall, aiming to release but preserve its nutritional and functional compounds (*7*). The antimicrobial activity of bee pollen can also be enhanced by processing techniques that break the complex pollen wall, thereby facilitating the release and activity of antimicrobial compounds. These techniques include mechanical, physical and enzymatic techniques, microbial fermentation, and their combinations. However, these treatments can also negatively affect the sensory properties of bee pollen, they can increase susceptibility to environmental factors, accelerate the degradation of bioactive compounds, increase the risk of microbial contamination, and consequently shorten the shelf life of bee pollen (*7*, *15*).

Numerous studies have confirmed the antimicrobial activity of bee pollen against various pathogenic bacteria and fungi (*1*-*3*, *5*, *8*-*10*), while other studies have not observed such effects (*16*, *17*). These discrepancies may result from differences in sampling, preparation and testing methodologies, but may also reflect the natural heterogeneity of bee pollen and the influence of its geographical and botanical diversity (*3*).

This review provides the first comprehensive analysis of studies on the antimicrobial activity of monofloral bee pollen. It identifies the most commonly investigated botanical species and targeted bacterial strains, and highlights key findings. The aim is to consolidate existing research on the antimicrobial activity of monofloral bee pollen, examining which species show the strongest antimicrobial effects, under which methodological approaches, and against which microbial targets. Furthermore, this review summarizes the processing methods applied in studies investigating the antimicrobial activity of bee pollen, comparing it before and after treatment. This part includes both, mono- and polyfloral samples, to emphasise the lack of research into pollen wall disruption methods and their potential to enhance the antimicrobial properties of bee pollen. Finally, it addresses gaps in combining novel processing methods with monofloral bee pollen and suggests directions for future research, supporting the ongoing effort to standardize bee pollen quality parameters, including its improved antimicrobial activity.

## ANTIMICROBIAL ACTIVITY

The antimicrobial activity of bee pollen results from the combined action of its active compounds. During pellet formation, bees introduce glucose oxidase, an enzyme that catalyzes the oxidation of glucose into gluconic acid and hydrogen peroxide (*18*). Hydrogen peroxide exerts bactericidal effects by damaging cell walls, proteins, and nucleic acids, while gluconic acid lowers the pH, creating an acidic microenvironment unfavourable for bacterial survival (*19*). Phenolic compounds, particularly flavonoids and phenolic acids, play a key role by disrupting bacterial cell membranes and triggering autolysis (*17*, *20*). Among flavonoids, tricetin, luteolin, quercetin and kaempferol are most commonly present, while cinnamic and ellagic acids stand out among phenolic acids for their potent antioxidant properties (*3*, *21*). Importantly, the antimicrobial activity of bee pollen depends more on the specific composition of phenolic compounds than on their total concentration. Extracts with relatively low overall phenolic compound content can still exhibit strong activity due to some bioactive molecules such as kaempferol 2-O-rhamnoside, quercetin 3-P-glucoside and isorhamnetin derivatives, which are frequently identified as key agents of microbial inhibition (*11*, *18*, *22*, *23*). Free fatty acids also contribute to antimicrobial activity by disrupting the electron transport chain and oxidative phosphorylation, inhibiting enzyme activity, and interfering with nutrient uptake. Capric, lauric, myristic, linoleic and linolenic acids are known for their antimicrobial effect, while palmitic, stearic and oleic acids do not exhibit such activity (*24*).

### Botanical origin

Advancements in molecular biology have introduced modern methods for determining the botanical origin of bee pollen, including profiling free amino acids, minerals, aromatic compounds, and especially DNA barcoding and next-generation sequencing. These methods offer high sensitivity but are limited by incomplete databases and costly equipment (*15*). Therefore, the botanical origin of bee pollen is most often determined through microscopic morphological and structural analysis of pollen grains, known as palynological analysis (*14*, *25*). This method requires a trained specialist to identify and classify grains based on characteristics such as size, shape, surface texture and aperture types. However, it is time-consuming and depends on the availability of a specialized palynologist (*13*). To classify pollen as monofloral bee pollen, it must contain 80 % or more pollen grains from a single plant species (*26*). The content of antimicrobial compounds in bee pollen is largely determined by its botanical origin (*27*). The number of studies on the antimicrobial activity of monofloral bee pollen extracts is increasing. However, only a limited number of studies investigating monofloral bee pollen extracts of the same botanical origin under comparable conditions are included in Table 1 (*5*, *28*-*35*).

**Table 1:** Review of studies investigating the antimicrobial activity of monofloral bee pollen extracts, with a focus on best activity reported for specific plant species

Author	Botanic species	Target microbial strain	Best antimicrobial activity *d*(inhibition zone)/mm, MIC/(mg/mL)
Fatrcová-Šramkova *et al.* (*28*)	Rapeseed (*B. napus*), opium poppy (*P. somniferum*), sunflower (*H. annuus*)	*L. monocytogenes* CCM 4699, *P. aeruginosa* CCM 1960, *S. aureus* CCM 3953, *S. enterica* CCM 4420, *E. coli* CCM 3988	mBPE (*B. napus*): 3.8 mm (*S. enterica*), 3.7 mm (*S. aureus*) eBPE (*P. somniferum*): 3.0 mm (*E. coli*) mBPE (*H. annuus*): 3.7 mm (*L. monocytogenes*) eMBE (*H. annuus*): 3.7 mm (*S. enterica*)
Khider *et al.* (*29*)	Maize (*Z. mays*), Egyptian clover (*T. alexandrinum*), date palm (*P. dactylifera*)	*E. coli* ATCC 25922, *S. *Enteritidis ATCC13076, *S. aureus* ATCC 8095, *L. monocytogenes* ATCC 15313, *L. bulgaricus* DSM 20081, *S. thermophilus* DSM 20617, *P. aeruginosa* PAO1	mBPE (*Z. mays*): 42 mm (*S. aureus*), 0.32 mg/mL (*E. coli, S. *Enteritidis*, L. monocytogenes, S. aureus*) mBPE (*T. alexandrinum*): 38 mm (*S. aureus*), 0.32 mg/mL (*E. coli, L. monocytogenes, S. aureus*) mBPE (*P. dactylifera*): 18 mm (*E. coli*), no MIC data
Mohdaly *et al.* (*5*)	Maize (*Z. mays*)	*L. monocytogenes* CIP 82.110, *S. aureus* CIP 76.25, *S. enterica* CIP 81.32, *E. coli* CIP 54.8	mBPE (*Z. mays*): 0.30 mg/mL (*L. monocytogenes*), 0.78 mg/mL (*S. aureus*)
Avşar *et al.* (*30*)	Chestnut (*C. sativa*)	*S. aureus* ATCC 6538, *E. faecalis* ATCC 51299, *B. cereus* 7064, MRSA, *E. coli* ATCC 11293, *K. pneumoniae,* *C. albicans* ATCC 14053, *C. krusei* ATCC 6258, *C. parapsilosis* ATCC 22019	mBPE (*C. sativa*): 23 mm (MRSA), 22 mm (*S. aureus*) Moderate anti-yeast activity
AbdElsalam *et al.* (*31*)	Egyptian clover (*T. alexandrinum*)	*S. aureus,* *P. aeruginosa,* *C. albicans, A. niger*	peBPE: 45 mm (*P. aeruginosa*), 38 mm (*S. aureus*) DCM BPE: 41 mm (*P. aeruginosa*), 33 mm (*S. aureus*) Moderate anti-yeast activity and weak antifungal activity
Spulber *et al.* (*32*)	Rapeseed (*Brassica* sp.), thistle (*Carduus* sp.), sunflower (*H. annuus*), plum (*Prunus* sp.), hawthorn (*C. monogyna*)	*E. coli* ATCC 25922, *P. aeruginosa* ATCC 27853, *S. aureus* ATCC 29213, *E. faecalis* ATCC 29212	eBPE (*Brassica* sp.): 20 mm (*S. aureus*) eBPE (*Carduus* sp.): 18 mm (*S. aureus*) eBPE (*H. anuus*): 15 mm (*E. faecalis)* eBPE (*Prunus* sp.): 17 mm (*E. faecalis*) eBPE (*C. monogyna)*: 18 mm (*S. aureus*)
Gabriele *et al.* (*33*)	Chestnut (*C. sativa*), blackberry/raspberry (*Rubus*), rockrose (*Cistus*)	*E. coli* ATCC 25922, *S.* Typhimurium ATCC 14028, *E. aerogenes* ATCC 13048, *S. aureus* ATCC 25923, *E. faecalis* ATCC 29212	eBPE (*C. sativa*): 10 mg/mL (*E. coli, S. aureus, S.* Typhimurium) eBPE (*Rubus*): 10 mg/mL (*E. faecalis, S. aureus*) eBPE (*Cistus*): 5 mg/mL (*E. faecalis, S. aureus*)
Sadeq *et al.* (*34*)	White savory (*M. fruticosa*), date palm (*P. dactylifera*), frangrant yarrow (*A. fragrantissima*)	*P. aeruginosa,* *E. coli* (ATB:57), *S. aureus,* *S. faecalis*	eBPE (*M. fruticosa*): 16.3 mm, 0.625 mg/mL (*S. faecalis*) eBPE (*A. fragrantissima*): 16.3 mm (*S. aureus*), 1.25 mg/mL (*S. faecalis*) eBPE (*P. dactylifera*): 14.7 mm, 0.15 mg/mL (*S. faecalis*)
Candan *et al.* (*35*)	Sunflower (*H. annuus*), opium poppy (*P. somniferum*)	*E. coli* ATCC 25922, *K. pneumoniae* ATCC 700603, *P. aeruginosa* ATCC 27853, *S. enterica* NCTC 12694, *B. cereus* ATCC 10876, *E. faecalis* ATCC 29212, *Listeria ivanovii* ATCC 19119, *L. monocytogenes* NCTC 10527, *S. aureus* ATCC 25923, *C. albicans* ATCC 10231	eBPE (*H. annuus*): 2.5 mg/mL (*S. aureus, L. ivanovii, E. faecalis, C. albicans*) eBPE (*P. somniferum*): 0.13 mg/mL (*E. faecalis*), 1.25 mg/mL (*L. ivanovii, C. albicans*), 2.5 mg/mL (*S. enterica, S. aureus, L. monocytogenes, K. pneumoniae*)

MIC=minimum inhibitory concentration, mBPE=methanolic bee pollen extract, eBPE=ethanolic bee pollen extract, peBPE=petroleum ether bee pollen extract, DCM BPE=dichloromethane bee pollen extract, MRSA=methicillin-resistant *S. aureus*

Rapeseed (*Brassica napus*), belonging to the Brassicaceae family, is one of the most important spring sources of nectar and pollen. When comparing the activity of rapeseed monofloral bee pollen extracts with those of other origins, they showed the activity against Gram-positive and Gram-negative bacteria, but it was weak, without significant differences with the use of different solvents (*28*). Later, a comparative study of six monofloral bee pollen extracts confirmed that rapeseed monofloral bee pollen extract had the strongest activity, especially against *Staphylococcus aureus* (*32*).

Opium poppy (*Papaver somniferum*), from the Papaveraceae family, showed limited antimicrobial activity in an early study (*28*). However, recent research reported strong activity against multiple bacterial and yeast pathogens (*35*). Further research is needed to confirm these findings.

The Asteraceae family, which includes sunflowers (*Helianthus annuus*), is an important source of pollen for bees. Sunflower bee pollen extracts have been frequently studied, revealing distinct phenolic profiles in methanolic and ethanolic extracts (*6*), as well as varying antimicrobial activity against *Paenibacillus larvae, Pseudomonas aeruginosa, Escherichia coli, Brochothrix thermosphacta* and *Enterococcus raffinosus* (*36*). Stronger antimicrobial activity has been observed against Gram-positive bacteria and fungi than against Gram-negative bacteria. This is consistent with the high lipid content of sunflower bee pollen, which may contribute to membrane-disrupting activity (*32*). In a recent study (*35*), sunflower monofloral bee pollen extract exhibited moderate antimicrobial activity against *S. aureus, Listeria ivanovii, Enterococcus faecalis* and *Candida albicans.*

Maize (*Zea mays*), from the Poaceae family, has shown strong antimicrobial activity against *S. aureus, E. coli* and *Salmonella*, suggesting the presence of potent bioactive compounds effective against both Gram-positive and Gram-negative bacteria (*5*, *29*). In the same study by Khider *et al.* (*29*), and in a more recent study (*34*), date palm (*Phoenix dactylifera*) monofloral bee pollen extracts were also investigated and exhibited activity against *E. coli* (*29*) and *S. faecalis* (*34*).

Chestnut (*Castanea sativa*) belongs to the Fagaceae family. Its pollen is characterized by a yellow-green colour and a rich content of bioactive compounds (*37*). Chestnut bee pollen has a stable phenolic fingerprint, dominated by phenolamines (N1,N5,N10-tricaffeoylspermidine), and consistently contains naringenin, which supports its strong antioxidant activity. In Slovenia, chestnut trees are widespread and serve as an important pollen source for bees (*38*). Studies of chestnut bee pollen have shown high amounts of polyphenols, flavonoids and anthocyanins. In antimicrobial assays, chestnut bee pollen inhibited the growth of *E. coli*, *Salmonella* Typhimurium and *S. aureus*. This activity is likely linked to the higher content of hydroxycinnamic acids and flavonoids (*33*). Methanolic extracts of chestnut bee pollens from nine locations in Turkey showed strong antimicrobial activity, particularly against *Micrococcus luteus* and *S. aureus*, including methicillin-resistant *S. aureus* (MRSA), and moderate efficacy against yeasts. *E. coli* exhibited high resistance, and no activity was observed against *K. pneumoniae* (*30*). The methanolic extract of chestnut bee pollen showed stronger antimicrobial activity than the ethanolic extract, especially against *S. aureus*. However, the ethanolic extract exhibited a broader antimicrobial spectrum, including activity against Gram-negative bacteria. Overall, chestnut monofloral bee pollen extracts demonstrate notable antimicrobial potential, influenced by the solvent type and the target bacteria (*30*, *33*).

Certain bee species are specialized in collecting pollen from plants of the Fabaceae family, which includes clover, beans and peas – plants also widely used as cover crops or forage (*35*). Bioactive compounds have been confirmed in members of the Fabaceae family, especially in red clover (*Trifolium pratense*). Red clover bee pollen extracts have significant antimicrobial activity, particularly against *S. aureus* and *P. aeruginosa*. Methanolic extracts of red clover demonstrated high efficacy against *S. aureus* and *E. coli*, and were substantially more effective than hexane-based bee pollen extracts (*29*). Similarly, red clover bee pollen extracts strongly inhibited *S. aureus* and *P. aeruginosa*, while antifungal activity against *Candida albicans* and *Aspergillus niger* was moderate or absent (*31*). Both studies highlight the effectiveness of ethanol and methanol-based red clover bee pollen extracts, attributing their activity to a high concentration of phenolic compounds, such as quercetin, kaempferol, caffeic acid and *p*-coumaric acid. These compounds exert their effects through multiple mechanisms, including disruption of bacterial cell membranes and inhibition of key enzymes, thereby explaining the broad-spectrum antimicrobial activity observed (*29*, *31*).

The Rosaceae family, including apple (*Malus domestica*), cherry (*Prunus avium*), plum (*Prunus domestica*), hawthorn (*Crataegus monogyna*) and various ornamental flowers, is also an important source of pollen for bees. Bee pollen extracts from *Prunus* species have shown moderate antimicrobial activity, primarily against *P. aeruginosa*, followed by *E. coli*, *E. faecalis* and *S. aureus.* This places *Prunus* sp. in the mid-range of effectiveness compared to other bee pollen extracts included in the study, which ranged from low activity (*H. annuus*) to higher activity (*Brassica* sp., *Carduus* sp.) (*32*). Bee pollen extracts from *Rubus* species (blackberries) exhibited weak activity, particularly compared to *Castanea* and *Cistus* species, and were only effective against Gram-positive bacteria (*33*). The ethanolic hawthorn bee pollen extract had moderate antimicrobial activity, comparable to that from thistle and rapeseed, and exceeded the activity observed in *Prunus* species and sunflower. Overall, hawthorn bee pollen showed a consistent inhibitory effect against *S. aureus* (*32*).

### Extraction solvents, target microorganisms and testing methods

The antimicrobial activity of bee pollen extracts depends strongly on the type and concentration of extraction solvent, which poses challenges for cross-study comparisons. Methanol and ethanol are the most frequently used and effective solvents, followed by water, hexane, butanol, and dimethyl sulfoxide (DMSO) (*3*, *10*). Ethanolic and methanolic monofloral bee pollen extracts have demonstrated broad-spectrum activity against *S. aureus*, *Candida glabrata*, *E. coli*, *Bacillus cereus*, *Bacillus subtilis*, *Salmonella *Enteritidis, *S. epidermidis*, *L. monocytogenes* and *P. aeruginosa* (*3*, *39*-*41*). Bee pollen extracts from *Trifolium* species varied in effectiveness, with ethanol, petroleum ether and dichloromethane extracts showing the strongest inhibition (*31*). These results highlight the importance of solvent choice as a primary determinant of the bioactivity of bee pollen extracts (Table 1) (*5*, *28*-*35*).

The antimicrobial activity of bee pollen extracts also depends on the target microorganism. Gram-positive bacteria are generally more sensitive than Gram-negative bacteria, which have complex lipopolysaccharide membranes and efflux pumps that confer resistance (*3*, *42*-*45*). Among the most frequently tested species, *S. aureus* is the most sensitive and thus serves as a reliable indicator of bee pollen extract efficacy, especially for methanolic, ethanolic (70 %) or dichloromethane extracts. *L. monocytogenes* shows variable sensitivity; methanolic or ethanolic extracts of sunflower, maize, clover, poppy and rapeseed monofloral bee pollen exhibited strong effects due to their content of flavonoids and other phenolic compounds, while others required higher concentrations for inhibition (*5*, *29*, *46*). *Enterococcus* sp. was highly resilient, with only modest inhibition reported (*30*, *33*, *36*).

Among Gram-negative species, results for *E. coli* range from strong to weak inhibition depending on pollen type: maize and clover monofloral bee pollen extracts showed notable effects, while those from sunflower, rapeseed, and plum were weaker (*29*, *32*, *36*, *47*). *Salmonella* generally showed low to moderate susceptibility, requiring high extract concentrations (*5*, *28*, *33*), and *P. aeruginosa* remained resistant, although butanol extracts enhanced its activity (*48*).

Antifungal studies have mainly targeted *Candida*, but bee pollen extracts have also shown inhibitory effects against *Aspergillus* and non-*Candida* yeasts (*22*, *31*, *39*, *47*, *49*). These effects are dose-dependent, with methanol/water extracts showing stronger effects than ethanol/water mixtures (*50*).

The results of antimicrobial activity are also influenced by the testing method. The two most common *in vitro* methods are the broth dilution method and the agar diffusion method (*3*). The agar diffusion method involves applying the extracts into wells or onto paper disks on inoculated agar, with antimicrobial activity measured by the diameter of the inhibition zone. This method is simple, cost-effective and reproducible, but less suitable for nonpolar extracts due to limited diffusion (*31*, *32*, *51*). The broth dilution method provides quantitative data by determining the minimum inhibitory concentration (MIC) and minimum bactericidal concentration (MBC) through serial dilutions in microplates, followed by measurement of microbial growth (*41*, *52*-*54*). Broth assays ensure immediate contact between the extracts and microorganisms, while agar assays rely on slower and uneven diffusion, which can be influenced by factors such as polarity, solubility, molecular size, or pore-blocking properties. Overall, the broth dilution method is considered more sensitive, whereas the agar diffusion method is valued for its reproducibility (*3*). A review of published studies on antimicrobial activity reveals that either method may be used, often with different measurement units and data presentation formats. These differences make direct comparison of results challenging.

### Processing of pollen

Pollen is enclosed by an outer wall called the exine, composed of the polysaccharide sporopollenin. This layer protects the pollen from physical and chemical stress and is coated with fats, carbohydrates, terpenoids and carotenoid pigments (*55*, *56*). Although the exine provides strong protection, it is thinner in regions known as germinal pores, which lead to the inner wall, the intine. The intine, primarily composed of pectin and cellulose, forms the final barrier before reaching the nutrient-rich cytoplasm (*55*).

To improve the bioavailability of nutrients and functional compounds, the pollen wall can be broken down using various processing methods (mechanical, physical, enzymatic, thermal, osmotic and fermentation, either individually or in combination). These treatments enhance nutrient release, digestion, and absorption in humans and animals (*57*), while also reducing allergenicity and improving antimicrobial activity (*7*, *15*).

#### Mechanical processing methods

Mechanical methods break down pollen walls using shear or friction forces. Equipment such as ball mills or high-speed shearing machines is commonly used. These methods are relatively simple and the equipment is cost-effective (*58*). Ball milling grinds materials using hard balls in a rotating container, applying compression and friction to reduce particle size, mix powders, and modify structures. In bee pollen research, it is used to break down tough pollen walls, thereby improving the accessibility of nutrients and bioactive compounds (*7*, *59*). However, the studies have shown that ball milling alone is less effective in breaking cell walls than ultrasonication. In contrast, the combination of ultrasonication and ball milling achieves significantly better results, enhancing the release of nutritional and bioactive compounds and providing the highest antioxidant and antimicrobial activity. This combined method produced the largest inhibition zones in tests against *E. coli*, *S. aureus*, *L. monocytogenes*, *C. albicans* and *Saccharomyces cerevisiae*, and also showed activity against *P. aeruginosa*, which was resistant to other bee pollen treatments. Moreover, compared to prolonged ball milling alone, this combined approach caused minimal degradation of the main bioactive compounds in bee pollen (*59*).

#### Drying

Due to its high water content (20–30 %), nutritional value and poor aseptic conditions in hives, bee pollen is susceptible to contamination. To prevent this, bee pollen is typically dried after collection to reduce moisture below 10 %, usually to 5–8 % (*60*-*62*), ensuring stability and quality during storage. Effective drying requires careful control of temperature, consideration of product characteristics, and treatment scope to prevent thermal degradation of sensitive components and to enhance subsequent processing of the pollen wall (*60*). The drying method and plant species significantly influence the outcome, as different species respond differently. Studies have examined the effects of drying on monofloral bee pollen compounds and properties from *Castanea sativa, Hedera helix, Salix* spp. (*63*), *Cistus ladanifer* (*64*), *Helianthus annuus* (*36*), *Rubus* spp., *Eucalyptus* spp*., Cistus* spp*., Cytisus* spp*., Echium* spp. and *Erica* spp. (*65*).

Traditional drying methods include sun drying, hot-air drying and the freeze-thaw method (*6*, *61*). However, to avoid many negative effects associated with traditional drying techniques, these have largely been replaced by industrial methods such as spray drying, freeze-drying, microwave drying and vacuum drying (*60*). Although high quality and yield are desired, drying methods are closely linked to cost. Sun drying is the cheapest but is rarely used due to environmental contamination, product loss, insect and bird interference, space requirements, process control difficulties, and odour issues (*66*). Hot-air and convection drying offer the best balance of cost, quality and speed (*67*). Higher temperatures shorten drying time but can reduce nutrient content, alter colour and negatively affect antioxidant, anti-inflammatory and antimicrobial activity (*63*, *68*, *69*). Drying at 40 °C has been shown to optimally preserve bee pollen quality (*67*).

Freeze-drying (lyophilization) is considered the most suitable method for preserving the colour, taste, bioactive compounds and biological properties of bee pollen (*60*, *61*). The process involves freezing at very low temperatures, followed by sublimation under reduced pressure, which disrupts the bee pollen wall and increases nutrient availability (*70*). Compared to hot-air drying, freeze-drying better preserves nutrients and bioactive compounds (*65*, *68*).

When studying the antimicrobial activity of bee pollen after drying, lyophilization consistently maintained stronger activity across most plant species. The lowest MICs were observed against *S. aureus* for methanolic bee pollen extracts from *Erica* species, followed by extracts from *Castanea sativa*, *Echium* and *Cistus* species (*65*). Table 2 (*22*, *36*, *65*, *71*) summarizes the studies that investigated the effects of different drying methods on the antimicrobial activity of bee pollen. Both monofloral bee pollen and polyfloral bee pollen are included in the review, due to the very limited number of studies investigating monofloral bee pollen so far.

**Table 2:** The influence of drying methods on antimicrobial activity of monofloral and polyfloral BPE

**Author**	**Botanic species**	**Target microbial strain**	**Processing method**	**Best antimicrobial activity** ***d*(inhibition zone)/mm, MIC/(mg/mL)**
Dias *et al*. (*65*)	*Erica* sp. *Rubus* sp. *C. sativa* *Cistus* sp. *Eucalyptus* sp. *Echium* sp.	*K. pneumoniae* ESA36, *P. aeruginosa* ESA38, *Enterococcus* ESA5, *S. aureus* ESA77, *C. parapsilosis* ESA70, *C. glabrata* ESA11	Drying (DRY), Lyophilization (LYO)	DRY m*BPE* (*Erica* sp.): 2.8 mg/mL (*S. aureus*) LYO mBPE (*Erica* sp.): 1.6 mg/mL (*S. aureus)* DRY mBPE (*C. sativa*): 3.9 mg/mL (*S. aureus*) LYO mBPE (*C. sativa*): 2.0 mg/mL (*S. aureus*) DRY mBPE (*Rubus* sp.): 6.0 mg/mL (*S. aureus*) LYO mBPE (*Rubus* sp.): 3.6 mg/mL (*S. aureus*)
Fatrcová-Šramková *et al*. (*36*)	Sunflower (*H. annuus*)	*E. coli* CCM 3988, *E. raffinosus* CCM 4216, *B. thermosphacta* CCM 4769, *P. larvae* CCM 4483, *P. aeruginosa* CCM 19 60, *A. flavus, A. fumigatus,* *A. niger, A. ochraceus, A. versicolor*	Drying (DRY), Freezing (FRZ), Lyophilization (LYO)	DRY eBPE: 2.6 mm (*P. larvae*), 2.7 mm (*E. coli*) FRZ eBPE: 2.7 mm (*E. coli*), 2.2 mm (*E. raffinosus*) LYO eBPE: 2.7 mm (*P. larvae*), 2.6 mm (*B. thermosphacta*) LYO eBPE: retained antifungal activity
De-Melo *et al*. (*22*)	Polyfloral	*E. coli* ATCC, ESA72, *Klebsiella* ATCC, ESA61, *S. pyogenes* ATCC, ESA12, *S. aureus* ATCC, ESA54, *C. albicans* ATCC, ESA109	Drying (DRY), Lyophilization (LYO)	LYO eMBPE: 2.1 mg/mL (*S. pyogenes* ATCC), 2.5 mg/mL (*S. pyogenes* ESA12) DRY eMBPE: 2.9 mg/mL (*S. pyogenes* ATCC), 3.2 mg/mL (*S. aureus* ATCC)
**Naibaho *et al*. (** ***71*** **)**	**Polyfloral**	***S. aureus* ATCC 25932,** ***E. coli* ATCC 8742,** ***S. epidermis* NN349,** ***Propionibacterium acnes* NN357**	**Drying (DRY),** **Chiller method** **(CH)**	**DRY eMBPE: 17 mm (*S. epidermis*), 0.125 mg/mL (*S. epidermis, S. aureus, P. acnes*)** **CH eMBPE: 16 mm (*S. epidermis*), 0.125 mg/mL (*S. epidermis, S. aureus, P. acnes*)**

MIC=minimum inhibitory concentration, mBPE=methanolic bee pollen extract, eBPE=ethanolic bee pollen extract, DRY=oven drying at 35–42 °C until 6–11 % moisture was reached in the product, CH=drying at 4 °C (14–22 days)

Lyophilization also proved superior to conventional drying in a study using polyfloral bee pollen, showing stronger activity against all studied bacteria and yeasts (*22*). For sunflower bee pollen extract, lyophilization and freezing were comparably effective when comparing dried, frozen and freeze-dried bee pollen against human and bee bacterial pathogens. Antifungal activity was highest in frozen pollen against *Aspergillus ochraceus* and in freeze-dried pollen against *A. niger*, with antibacterial activity stronger than antifungal activity (*36*). When comparing drying methods using a chiller (4 °C) *versus* oven drying, antimicrobial activity was higher in bee pollen processed with the 4 °C chiller method (*71*). Despite its effectiveness, freeze-drying has limitations. It is relatively expensive and time-consuming, making it less suitable for industrial use where mass production is required (*60*, *61*).

Microwave drying uses electromagnetic waves for efficient heating and dehydration, preserving bioactive compounds while minimizing thermal stress (*60*, *61*). Controlled power and moisture levels are essential to ensure product quality. At lower power, microwave drying produces nutritionally rich bee pollen faster than other methods (*61*). To the best of our knowledge, there are no studies on the antimicrobial activity of bee pollen before and after microwave drying. Despite this gap in the literature, existing studies indicate that the antimicrobial activity of bee pollen is generally retained following various drying techniques, with lyophilization being the most effective. The choice of drying method, in combination with the plant species, also has a significant influence, as not all species respond equally to the same drying technique (*36*, *65*). However, only a limited number of studies have examined the effect of drying on the antimicrobial activity of bee pollen – particularly monofloral bee pollen. This gap should be addressed in future research focused on developing quality parameters for monofloral bee pollen.

#### Ultrasound

Ultrasound is a cost-effective, simple and energy-efficient method for disrupting the walls of bee pollen. It increases membrane permeability and induces cavitation, which fragments cellular structures (*72*). Low-power ultrasound is mainly used for monitoring, while high-power ultrasound causes structural changes. As a non-thermal process, it preserves heat-sensitive compounds (*73*). However, prolonged use can reduce enzyme activity and efficiency (*74*).

Ultrasonication significantly enhances the bioactivity of bee pollen. After 5 h of treatment, increases were observed in 2,2-diphenyl-1-picrylhydrazyl (DPPH), 2,2′-azino-bis(3-ethylbenzothiazoline-6-sulfonic acid (ABTS), total phenolic and total flavonoid content. Similar enhancements were observed with supercritical fluid extraction (*75*). Combining ultrasonication with complementary methods, such as high shear or enzymatic treatment, further disrupts pollen walls, enhances the release of bioactive compounds, improves protein yield and solubility, and boosts functional properties like digestibility, emulsifying capacity and gelling ability (*57*, *76*).

The antimicrobial activity of treated bee pollen showed high variability. Some studies reported no antimicrobial activity against several enteric pathogens, with either conventional or sonication-based extraction (*77*), while others observed enhanced antioxidant activity (*75*, *78*, *79*), and both antioxidant and antimicrobial activity – particularly against *S. aureus* (*59*, *80*). *S. aureus* appears especially sensitive to ethanolic bee pollen extract when processed with ball milling, ultrasonication, or their combination, unlike *P. aeruginosa*, which shows little to no inhibition. When comparing ball milling and ultrasonication, the latter demonstrated a stronger cell wall-breaking effect. However, the combination of both methods proved most effective, resulting in the highest antioxidant and antimicrobial activities (*59*).

Recent advances in sustainable biotechnology have introduced innovative processing methods for bee pollen. Ultrasound and green extraction methods, such as deep eutectic solvents (DES) and supercritical fluid extraction, enhance the antioxidant and antimicrobial activity, and the functional properties of bee pollen. These eco-friendly techniques improve the yield of bioactive compounds and produce high-value ingredients for food supplements (*59*, *75*, *80*, *81*). DES-treated bee pollen extracts exhibited strong antimicrobial activity, especially against Gram-positive bacteria like *S. aureus.* Antifungal activity was limited, indicating lower effectiveness against yeast-like fungi. Broad-spectrum antibacterial activity was observed across all tested strains, with extracts at molar ratios of 1:1.5 and 1:2 showing slightly higher inhibition than 1:1, suggesting that the hydrogen bond donor (HBD) to hydrogen bond acceptor (HBA) ratio influences antibacterial potency (*80*). To date, no published research has directly compared the antimicrobial activity before and after ultrasound treatment of monofloral bee pollen.

#### Fermentation

Microbial fermentation is an efficient method for transforming basic food ingredients, enhancing unique flavours and other sensory properties, improving nutritional profiles by degrading ant-nutrients and increasing beneficial nutrients, and extending shelf-life (*82*). *In vitro* fermentation of bee pollen, modelled after its natural transformation into bee bread in the hive, can enhance its antimicrobial activity and functional properties (*15*, *83*). In the absence of oxygen, yeasts, lactic acid bacteria or a combination of both break down the multilayered pollen wall and degrade macromolecules into smaller, more bioavailable compounds. This process increases nutrient content and improves the accessibility of bioactive compounds (*7*, *20*, *84*, *85*). Additionally, lactic acid bacteria produce bacteriocins, substances that disrupt bacterial membranes, which are effective against pathogens such as *L. monocytogenes*, *Listeria innocua*, *S. aureus*, *E. coli*, *M. luteus*, *P. aeruginosa*, *B. subtilis*, *S.* Enteritidis and *S.* Typhimurium (*86*).

Across multiple studies, fermentation consistently enhanced the antimicrobial activity of bee pollen extract. Both spontaneous and induced bacterial fermentation have been shown to increase antimicrobial effects, with Gram-positive bacteria generally being more sensitive than Gram-negative strains (*86*-*88*). Kaškoniene *et al.* (*87*) confirmed improved antimicrobial activity, showing an almost twofold increase in inhibition zones for *M. luteus* after spontaneous fermentation. Urcan *et al.* (*88*) reported that ethanolic bee pollen extracts showed a substantial reduction in MIC values after fermentation, nearly a twofold decrease for *S. aureus*, *E. faecalis*, *E. coli* and *P. aeruginosa*, indicating strongly improved antimicrobial potency against both Gram-positive and Gram-negative bacteria. Similar trends were observed in methanolic bee pollen extracts, with inhibition zones moderately increased after spontaneous and bacterial-induced fermentation of bee pollen (*86*).

#### Enzymatic hydrolysis

Enzymatic hydrolysis enhances nutrient release and bioactivity in bee pollen by breaking down polysaccharides, proteins and other macromolecules (*60*, *89*). This improves digestibility, permeability and bioavailability, resulting in increased antioxidant and antimicrobial activity (*90*). The efficiency of enzymatic hydrolysis depends on several factors, including enzyme type, concentration, pH, temperature, and duration of hydrolysis (*91*). Controlled hydrolysis can produce bioactive peptides with strong angiotensin-converting enzyme (ACE)-inhibitory and antioxidant activity (*92*), as well as improved protein solubility and functionality compared to physical methods (*76*). Proteases (*e.g.* Protamex) enhance the release of proteins, phenolic compounds and flavonoids, while cellulases, pectinases and carbohydrases facilitate the release of bioactive compounds and improve functional properties (*7*, *89*, *93*). Combined methods, such as enzymatic hydrolysis with ultrasound or freeze-thaw cycles, can further enhance yield and functionality (*58*, *76*).

Only a few studies have investigated the impact of enzymatic hydrolysis on the antimicrobial activity of bee pollen. Available research shows that enzymatically hydrolyzed bee pollen exhibits increased effectiveness against both Gram-positive bacteria (*S. aureus*, *L. monocytogenes*) and Gram-negative bacteria (*S.* Enteritidis, *S.* Typhimurium) (*86*, *89*).

Gram-positive bacterial strains showed greater sensitivity to bee pollen extracts of both natural and enzymatically hydrolyzed bee pollen, likely due to differences in bacterial cell wall structure. In the study by Damuliene *et al.* (*89*), the first published investigation optimizing enzymatic hydrolysis parameters such as duration, enzyme concentration and substrate pH, the antimicrobial activity before and after hydrolysis was strongly correlated with total phenolic content, total flavonoids and antioxidant activity. This confirms that both the composition and quantity of bioactive compounds significantly influence the antimicrobial properties of bee pollen. In this study, cellulase and Viscozyme® L achieved the best results (*89*).

Further research on combined pollen wall treatment methods, including fermentation and enzymatic hydrolysis, showed that both spontaneous and induced bacterial fermentation significantly enhanced the antimicrobial activity of bee pollen. Induced bacterial fermentation with *L. rhamnosus* produced the highest increase. Enzymatic hydrolysis further boosted antimicrobial activity more consistently than fermentation, with Clara-diastase, Viscozyme® L, and cellulase providing the greatest improvements (*86*).

To date, no studies have investigated the effects of pollen processing methods such as ball milling, ultrasonication, fermentation and enzymatic hydrolysis on the antimicrobial activity of monofloral bee pollen. However, given the growing number of studies on the antimicrobial properties of monofloral bee pollen, such research is expected in the near future. Table 3 (*59*, *80*, *86*-*89*) summarizes the current information about the influence of processing on the antimicrobial activity of polyfloral bee pollen, showing the highest and lowest observed activity.

**Table 3:** The effect of different pollen wall disruption processing methods on antimicrobial activity of polyfloral BP

Author	Target microbial strain	Processing method	Antimicrobial activity *d*(inhibition zone)/mm, MIC/(mg/mL), before and after processing
Kaškoniene *et al.* (*87*)	*M. luteus* ATCC 4698, *S. aureus* ATCC 6538, *E. coli* ATCC 8739	Fermentation (F)	before F: 1.9 mm (*E. coli*), 7.2 mm (*M. luteus*) after spontaneous F: 3.3 mm (*E. coli*), 14.7 mm (*M. luteus*) after induced F: 3.8 mm (*E. coli*), 12.8 mm (*M. luteus*)
Çelik *et al.* (*80*)	*B. cereus* BC 6830, *B. cereus* ATCC 14579, S. *mutans* ATCC 35668, *S. aureus* NCTC 10788, BC 7231 *Acinetobacter baumannii* BHP1101 *E. coli* NCTC 9001, P. *aeruginosa* NCTC 12924, *S*. Typhimurium RSSK95091, Y*ersinia enterocolitica* ATCC 27729, *C. albicans* SB1, *C. glabrata* SB5, *C. krusei* SB8, *C. albicans* ATCC 10231	Sonication (SON) with deep eutectic solvents (DESs)	No data before treatment eBPE: 24 mm (*B. cereus* ATCC 14579), 7 mm (all *Candida* spp. tested, *S. cerevisiae*)
Chehraghi *et al.* (*59*)	*E. coli,* *S. aureus,* *L. monocytogenes,* *P. aeruginosa,* *C. albicans, S. cerevisiae*	Ball milling (BM), Ultrasonication (uSON)	eBPE: 9 mm (*L. monocytogenes*), 0 mm (*S. aureus, S. cerevisiae, E. coli*) eBPE after BM: 18 mm (*S. aureus*), 0 mm (*P. aeruginosa*) eBPE after uSON: 20 mm (*S. aureus*), 0 mm (*P. aeruginosa*) eBPE after BM/uSON: 23 mm (*S. aureus*), 8 mm (*P. aeruginosa*)
Damulienė *et al*. (*89*)	*S. aureus,* *L. monocytogenes,* *S. *Enteritidis*, * *S. *Typhimurium	Enzymatic hydrolysis (EH)	mBPE before EH: µg CEF/mL from 14 µg CEF/mL (*S. aureus*) to 7 µg CEF/mL (*S.* Typhimurium) mBPE after EH: µg CEF/mL from 30.54 µg CEF/mL (S. *aureus*) with celullase to 4.70 µg CEF/mL (*S.* Typhimurium) with amyloglucosidase
Urcan *et al.* (*88*)	*S. aureus* (ATCC 25923) *E. faecalis* (ATCC 29212) *E. coli* (ATCC 25922) *P. aeruginosa* (ATCC 27853) *C. albicans* (ATCC 10231) Bacteria for fermentation: *L. plantarum, L. acidophilus*	Fermentation (F)	eBPE F: 0.78 mg/mL (*S. aureus, E. faecalis*), 25 mg/mL (*P. aeruginosa*) eBPE after F: 0.38 mg/mL (*S. aureus, E. faecalis, E. coli*), 2.50 mg/mL (*P. aeruginosa*)
Damulienė *et al*. (*86*)	*S. aureus,* *L. monocytogenes,* *S.* Enteritidis, *S. *Typhimurium	Fermentation (F), Enzyme hydrolysis (EH)	mBPE before F/EH: 8 mm (*S. aureus*), 4 mm (*S*. Typhimurium) mBPE after spontaneous F: 11.3 mm (*S. aureus*), 5.2 mm (*S*. Typhimurium) mBPE after induced F: 13.2 mm (*S. aureus*), 6.1 mm (*S*. Typhimurium) mBPE after EH: 20.7 mm (*S. aureus*) with cellulase, 5.3 mm (*S*. Typhimurium) with amyloglucosidase

MIC=minimum inhibitory concentration, eBPE=ethanolic bee pollen extract, mBPE=methanolic bee pollen extract

## CONCLUSIONS

Comparing published data and interpreting results across different studies is challenging, particularly when applying these findings in practical contexts, due to the many factors that contribute to the high variability of the antimicrobial activity of bee pollen. This variability is primarily determined by the chemical composition of bee pollen, which depends on its botanical origin and the corresponding concentrations of phytochemicals. The composition is also influenced by geographical location, habitat, seasonal and weather conditions, bee subspecies and beekeeping practices. Sampling, storage and extraction methods, especially the use of different solvents, further affect the quality and comparability of results, even when samples originate from the same plant species.

A review of studies on the antimicrobial activity of monofloral bee pollen extracts reveals a growing research trend in this area. The most frequently investigated botanical species include sunflower, oilseed rape, clover and chestnut. Antimicrobial activity of monofloral bee pollen extracts has been examined against various microbial targets, most commonly *S. aureus* and enteric pathogens. In general, Gram-positive bacteria demonstrated greater sensitivity to bee pollen extracts than Gram-negative bacteria, with *S. aureus* consistently confirmed as susceptible.

Future research should aim to clarify the effects of both botanical origin and processing methods on pollen wall integrity, compound bioavailability and antimicrobial activity, particularly focusing on monofloral bee pollen, its bioactive constituents, and species-specific quality standards. The increasing number of monofloral bee pollen studies is encouraging. However, further research is needed to provide new insights into its bioactive potential, especially when combined with innovative processing techniques. Although monofloral bee pollen production remains limited, it is gaining attention due to market demand for traceable, high-quality, standardized products. Additionally, the development of standardized polyfloral bee pollen blends from various monofloral bee pollens could enable beekeepers to offer a diverse selection of specific bee pollen types with uniform composition and biological activity — key factors in producing high-quality bee pollen products for applications in the food industry and human nutrition.
